# Modification of the 8th American Joint Committee on Cancer staging system for gallbladder carcinoma to improve prognostic precision

**DOI:** 10.1186/s12885-020-07578-7

**Published:** 2020-11-23

**Authors:** Wei Jiang, Bingqing Zhao, Yongcheng Li, Dunfeng Qi, Daxing Wang

**Affiliations:** 1 Department of General Surgery, Tianjin First Central Hospital, Tianjin, 300192 China; 2 Department of Surgery, Tianjin Second People’s Hospital, Tianjin, 300192 China; 3 Department of Medical Oncology, Xuzhou Central Hospital, The Affiliated Xuzhou Hospital of Medical College of Southeast University, Xuzhou, 221009 China; 4 Department of Hepatic-Biliary-Pancreatic-Splenic Surgery, Xuzhou Central Hospital, The Affiliated Xuzhou Hospital of Medical College of Southeast University, Xuzhou, 221009 China; 5 Department of General Surgery, The People’s Hospital of Huaiyin Jinan, Jinan, 250021 China

**Keywords:** Gallbladder carcinoma, SEER, Overall survival, AJCC, Stage, Prognosis

## Abstract

**Background:**

The 8th edition of the American Joint Committee on Cancer (AJCC) staging system for gallbladder carcinoma (GBC) came into force since 2018. However, the prognostic precision of this staging system has not been properly assessed. This study aimed to evaluate the latest staging system and suggest modifications to improve its prognostic precision.

**Methods:**

Data of patients with GBC was included from the Surveillance, Epidemiology and End Results (SEER) database (2004–2015) and multicenter database (2010–2017). Baseline clinicopathologic characteristics were recorded including age, sex, race, grade, T category, N category, M category and stage. The Kaplan-Meier method was used to plot survival functions. The prediction power of the AJCC 8th edition and its modified version were evaluated using the concordance index (C-index).

**Results:**

A total of 2779 GBC patients were included in the SEER database and 591 were collected from multicenter database. While no significant difference in survival of patients was observed between stages IVA and IVB using the 8th AJCC staging system (*p* > 0.05), the prognosis of stage IIIA showed a slightly better outcome than stage IIIB (*p* = 0.046) in the SEER database. In the multicenter database, there was no significant difference between stage IIIA and stage IIIB (*p* > 0.05). Similarly, no significant difference in the survival of patients between stages IIIA and IIIB was observed when M0 patients with at least 6 lymph nodes (LNs) were analyzed (*p* > 0.05) for both SEER and multicenter database. On the other hand, a modified staging system was able to stratify patients from stage IIIA, stage IIIB and stage IV (*p* < 0.001). For the SEER database, the C-indexes of 8th AJCC staging system and that of its modified version were 0.709 and 0.742, respectively. For the multicenter database, the C-index of 8th AJCC staging system and that of our modified version were 0.635 and 0.679, respectively.

**Conclusions:**

The modified 8th staging system proposed in this study can improve the prognostic precision of the 8th AJCC staging system for GBC. We therefore suggest including these modifications in the next update of AJCC staging system for GBC.

**Supplementary Information:**

The online version contains supplementary material available at 10.1186/s12885-020-07578-7.

## Background

Gallbladder carcinoma (GBC) is a relatively rare malignancy with an incidence rate of 2.5 in 100,000 individuals. A total of 11,740 biliary malignancy cases, including GBC, were reported in the United States in 2017, and 52,800 cases were reported in China in 2015 [1, 2]. Patients are often diagnosed at advanced stages of GBC due to the elusive signs and symptoms of the disease [3–5].

Despite improvements in the chemotherapy and surgery of the disease, GBC is still associated with poor outcomes. Indeed, the 5-year survival rate of GBC remains less than 5% [5], which requires multi-aspect three-dimensional treatment plans for GBC patients in order to manage their disease properly. The 8th edition of the American Joint Committee on Cancer (AJCC) staging system, the most authoritative prognostic manual for malignancies, was released in 2016 and has come into force in 2018 [6]. Two noticeable modifications that provided new definitions for T and N categories were included in the latest AJCC staging system for GBC.

The T2 category has now been divided according to the anatomical location of the gallbladder into T2a (peritoneal side) and T2b (hepatic side), which points to the importance of the tumor location as a key prognostic factor. On the other hand, the N category has been divided according to the number of metastatic LNs, instead of the anatomic location, into N1 (1–3 positive LNs) and N2 (≥4 positive LNs) categories. It was also recommended that at least 6 LNs should be examined for prognosis, which means that patients with the previous N1 category (metastatic LNs along the cystic duct, common bile duct, hepatic artery, and/or portal vein) will be reclassified as N2 category (stage IVB) if they have four or more metastatic LNs. Similarly, some patients with the previous N2 category (metastatic LNs to periaortic, pericaval, superior mesenteric artery, and/or celiac artery) will be re-classified into N1 category if they have less than four metastatic LNs.

It is still unclear whether the new re-classification has a better prognostic power than the previous staging system. For example, the 8th AJCC staging system cannot adequately categorize the tumor’s biologic potential and the prognostic outcomes for pancreatic ductal adenocarcinoma (PDAC) patients [6]. The same issue was observed for gastric adenocarcinoma [7]. In this regard, an update of the staging system is often proposed. Therefore, we aimed to assess the prognostic precision of the 8th AJCC staging system for GBC and suggest some modifications by analyzing patient’s data extracted from the Surveillance, Epidemiology, and End Results (SEER) database and multicenter database.

## Methods

### Patients

Patients diagnosed with GBC between 2004 and 2015, with tumor site recoded as gallbladder according to the International Classification of Diseases for Oncology (ICD-O-3/WHO 2008), were selected from the SEER database. The validation data were from Tianjin First Central Hospital and Tianjin Second People’s Hospital (2010–2017). The inclusion criteria were patients that were: (1) aged 18 years or older; (2) showed positive histology report; (3) diagnosed as first primary malignancy; (4) precisely categorized as T and M category according to the 8th edition of AJCC staging system; (5) classified as M0 with a definite number of positive lymph nodes (LNs). Patients with unknown survival time or survival status were excluded from the study. Baseline clinicopathologic characteristics were recorded, including age, sex, race, grade, chemotherapy, radiotherapy, T category, N category, M category and stage. On the other hand, T2 patients were regarded as a single population in our analysis, instead of T2a and T2b, because data from 2004 to 2015 lacked relevant information related to the 8th AJCC staging system. Follow-up time ranged from 0 to 142 months with a median time of 17 months in the SEER database and from 0 to 95 months with a median time of 14 months in the multicenter database.

### Statistical analysis

Overall survival (OS) was the primary study endpoint and death from any cause was considered as an event. The median OS was calculated using 95% confidence interval (95% CI) separately for each of the T, N and M categories when they were considered as individual groups. Kaplan-Meier survival curve was used to plot survival functions over time. The concordance index (C-index) was used to evaluate the stratification power of the 8th AJCC edition and that of our modified version. Statistical analysis was performed using the IBM SPSS Statistics 22. A *p*-value < 0.05 was used as a cutoff of statistical significance.

## Results

### Patient characteristics

Data for 2779 GBC patients diagnosed between 2004 and 2015 were downloaded from the SEER database, including 298 stage I cases, 592 stage II cases, 326 stage IIIA cases, 897 stage IIIB cases, 80 stage IVA cases and 586 stage IVB cases (Table 1). Most of the patients in the cohort were female (70.0%) and White (75.6%) subjects. The age of the patients ranged between 18 and 101 years with a median age of 67 years. Tumors were classified as well/moderate differentiated tumors in 1525 patients and poor/undifferentiated tumors in 1038 patients. One thousand one hundred eighty-three cases (42.6%) received chemotherapy and 703 cased (26.0%) received radiotherapy. The number of LN evaluation ranged from 1 to 40 with a median number of 2. Two thousand one hundred thirty cases (78.8%) had less than six LNs resected, while only 573 cases (21.2%) had six or more LNs resected. The number of positive LNs ranged from 0 to 22 with the median number of 1. According to the 8th edition of AJCC staging system, 1258 cases belonged to the N1 category, whereas only 140 cases belonged to the N2 category. The survival curve of each N category was provided in Supplement 1.

**Table 1:** Baseline Characteristics of Patients with Gallbladder Carcinoma from the SEER and Multicenter Database

Variables	Frequency (%)	
	SEER (2779)	Multicenter (591)
**Age**		
< 65	1130 (40.7%)	314 (53.1%)
≥ 65	1649 (59.4%)	277 (46.9%)
**Sex**		
Male	835 (30.0%)	209 (35.4%)
Female	1944 (70.0%)	382 (64.6%)
**Race**		
White	2100 (75.6%)	0
Black	348 (12.5%)	0
Others	331 (11.9%)	591 (100%)
**Vital Status**		
Dead	1724 (62.0%)	436 (73.8%)
Alive	1055 (38%)	155 (26.2%)
**Grade**		
Well/moderate	1525 (54.9%)	360 (60.9%)
Poor/undifferentiated	1038 (37.3%)	231 (39.1%)
Unknown	216 (7.8%)	0
**T category(8th)**		
T1	377 (13.6%)	91 (15.4%)
T2	1187 (42.7%)	264 (44.7%)
T3	1083 (39.0%)	210 (35.5%)
T4	132 (4.7%)	26 (4.4%)
**N category(8th)**		
N0	1381 (49.7%)	337 (57.0%)
N1	1258 (45.3%)	219 (37.1%)
N2	140 (5.0%)	35 (5.9%)
Median (range)	1 (0–22)	2 (0–9)
**No. of LN evaluation**^**a**^		
< 6	2130 (78.8%)	413 (69.9%)
≥ 6	573 (21.2%)	178 (30.1%)
Median (range)	2 (1–40)	2 (1–24)
**M category(8th)**		
M0	2290 (82.4%)	483 (81.7%)
M1	489 (17.6%)	108 (18.3%)
**TNM Stage(8th)**		
I	298 (10.7%)	57 (9.6%)
II	592 (21.3%)	149 (25.2%)
IIIA	326 (11.7%)	62 (10.5%)
IIIB	897 (32.3%)	174 (29.4%)
IVA	80 (2.9%)	18 (3.0%)
IVB	586 (21.1%)	132 (22.3%)
Chemotherapy		
Yes	1183 (42.6%)	317 (53.6%)
No/unknown	1596 (57.4%)	274 (46.4%)
Radiotherapy		
Yes	723 (26.0%)	184 (31.1%)
No	2056 (74.0%)	407 (68.9%)

^a^76 cases missing in the SEER database

In total, 591 patients from multicenter were included, including 57 stage I cases, 149 stage II cases, 62 stage IIIA cases, 174 stage IIIB cases, 17 stage IVA cases and 132 stage IVB cases. The number of LN evaluation ranged from 1 to 24 with a median number of 2. Four hundred thirteen cases (69.9%) had less than six LNs resected, while only 178 cases (30.1%) had six or more LNs resected.

The median OS was calculated separately for each of the T, N and M categories when they were considered as individual groups (Table 2). The median OS of T3N1M0 was significantly shorter than that of T1N1M0 and T2N1M0 (*p* < 0.001) despite that it was classified as stage IIIB. Similarly, although stage IVB included T1N2M0, T2N2M0, T3N2M0 and T4N2M0, the four categories showed different median OS, where the median OS of T4N2M0 was merely 4 months, while that of T1N2M0 and T2N2M0 was approximately 20 months, which was longer than the 13-months median OS of T3N1M0 (SEER, *p* < 0.001). The data from the multicenter indicated a similar result. These results suggest that the latest AJCC staging system should be improved to obtain a better prognostic accuracy.

**Table 2:** Median OS of each TNM group

Stage (8th)	Group	Median OS (95% CI)		Stage (8th modified)
		SEER	Multicenter	
I	T1N0M0	95 (73.4–116.6)	74 (53.2–115.7)	I
II	T2N0M0	79 (56.4–101.6)	55 (39.4–70.6)	II
IIIA	T3N0M0	20 (16.8–23.2)	23 (13.5–34.8)	IIIA
IIIB	T1N1M0	17 (11.4–22.6)	20 (12.6–27.1)	IIIA
IIIB	T2N1M0	24 (19.3–28.7)	19 (12.2–28.0)	IIIA
IIIB	T3N1M0	13 (11.4–14.6)	15 (10.1–21.7)	IIIB
IVA	T4N0M0	12 (8.1–15.9)	12 (7.9–17.2)	IIIB
IVA	T4N1M0	10 (7.2–12.8)	12 (7.4–16.9)	IIIB
IVB	T1N2M0	21 (0–48.4)	22 (15.5–31.3)	IIIA
IVB	T2N2M0	20 (13.1–26.9)	17 (10.7–26.6)	IIIA
IVB	T3N2M0	11 (9.2–12.8)	13 (8.4–18.0)	IIIB
IVB	T4N2M0	4 (1.1–6.9)	9 (7.1–11.9)	IV
IVB	T*any*N*any*M1	8 (7.2–8.8)	7 (5.4–10.1)	IV

*OS* overall survival, *CI* confidence interval

### Comparison between the 8th staging system and the modified staging system

**Fig. 1:**
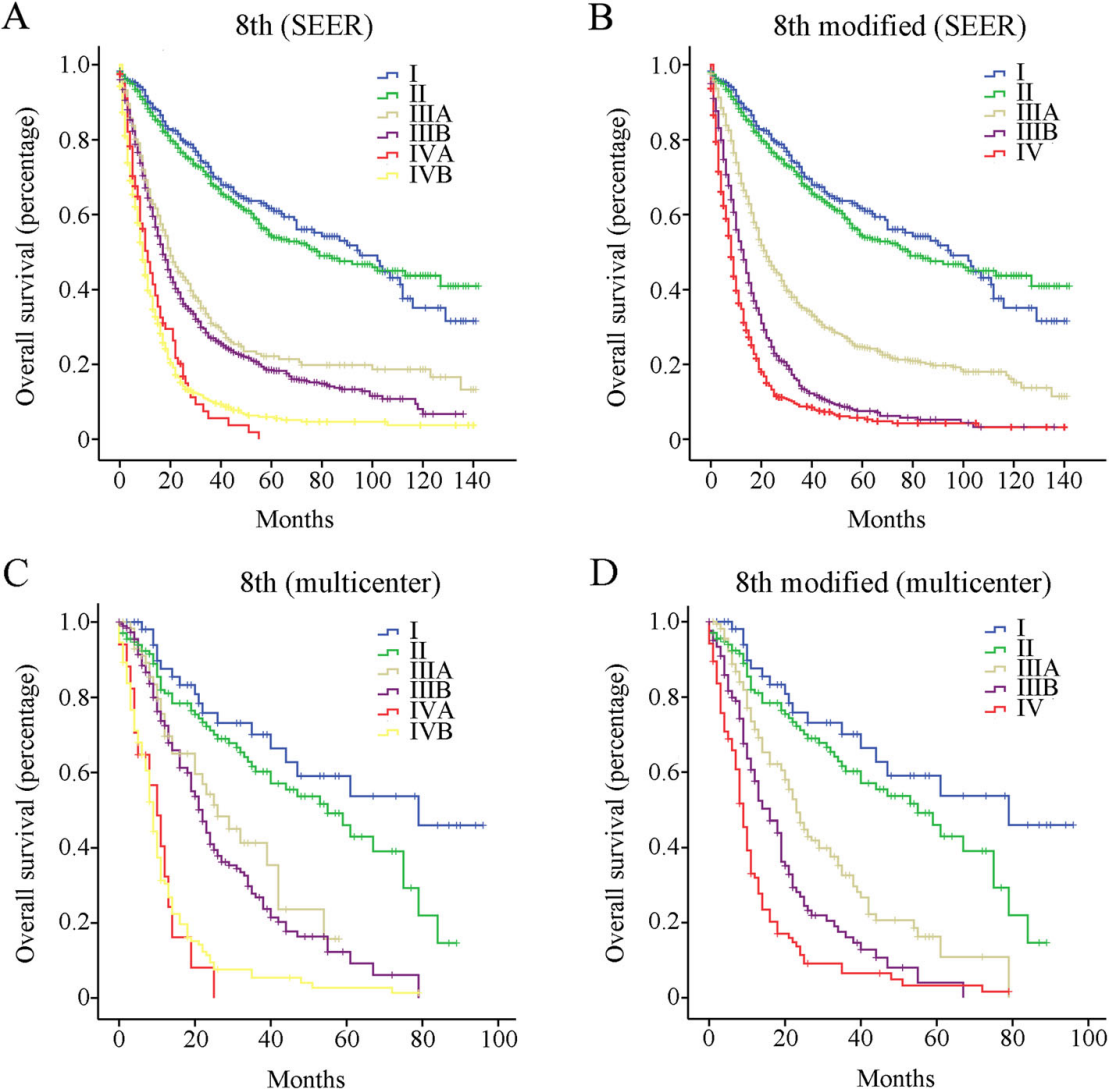
Overall survival (OS) analysis of the whole Gallbladder cancer (GBC) cohort. **a** OS of GBC patients from the SEER database using the 8th edition of AJCC staging system. **b** OS of GBC patients from the SEER database using the modified 8th edition of AJCC staging system. **c** OS of GBC patients from the multicenter database using the 8th edition of AJCC staging system. **d** OS of GBC patients from the multicenter database using the modified 8th edition of AJCC staging system

Patients were stratified according to the 8th edition of AJCC staging system, and survival curves were plotted using the Kaplan-Meier estimator (SEER, Fig. 1a; multicenter, Fig. 1c). No significant difference in OS was observed between stage IVA and IVB (*p* > 0.05). On the other hand, the OS of stage IIIA patients (median OS of 20 months) was slightly shorter than that of stage IIIB (median OS of 17 months, *p* = 0.046) in the SEER. Similar result was observed in the multicenter database although the difference was not significant (p > 0.05).

The staging system was then modified to include the following groups (Table 3): stage I (T1N0M0), stage II (T2N0M0), stage IIIA (T3N0M0, T1-2N1-2M0), stage IIIB (T3N1-2 M0, T4N0-1 M0) and stage IV (T4N2M0, T*any*N*any*M1). As shown in Fig. 1b and d, a significant survival difference was observed between stage IIIA, IIIB and IV (*p* < 0.001) for both SEER and multicenter database. The predictive abilities of our modified version and that of the AJCC system were assessed using the C-index. For the SEER database, the C-index of 8th AJCC staging system was 0.682 (95% CI, 0.645–0.719), while that of our modified staging system was 0.697 (95% CI, 0.660–0.734). For the multicenter database, the C-index of 8th AJCC staging system was 0.629 (95% CI, 0.594–0.651), while that of our modified staging system was 0.667 (95% CI, 0.634–0.690).

**Table 3:** AJCC Prognostic Staging Groups

TNM (8th)	Stage (8th)	Stage (modified 8th)	TNM (modified 8th)
T1N0M0	**I**	**I**	T1N0M0
T2N0M0	**II**	**II**	T2N0M0
T3N0M0	**IIIA**	**IIIA**	T3N0M0, T1-2N1-2M0
T1–3N1M0	**IIIB**	**IIIB**	T3N1-2 M0, T4N0-1 M0
T4N0-1 M0	**IVA**	**IV**	T4N2M0, T*any*N*any*M1
T*any*N2M0	**IVB**		
T*any*N*any*M1			

*AJCC* American Joint Committee on Cancer, *T* primary tumor, *N* lymph nodes, *M* distant metastasis

**Fig. 2:**
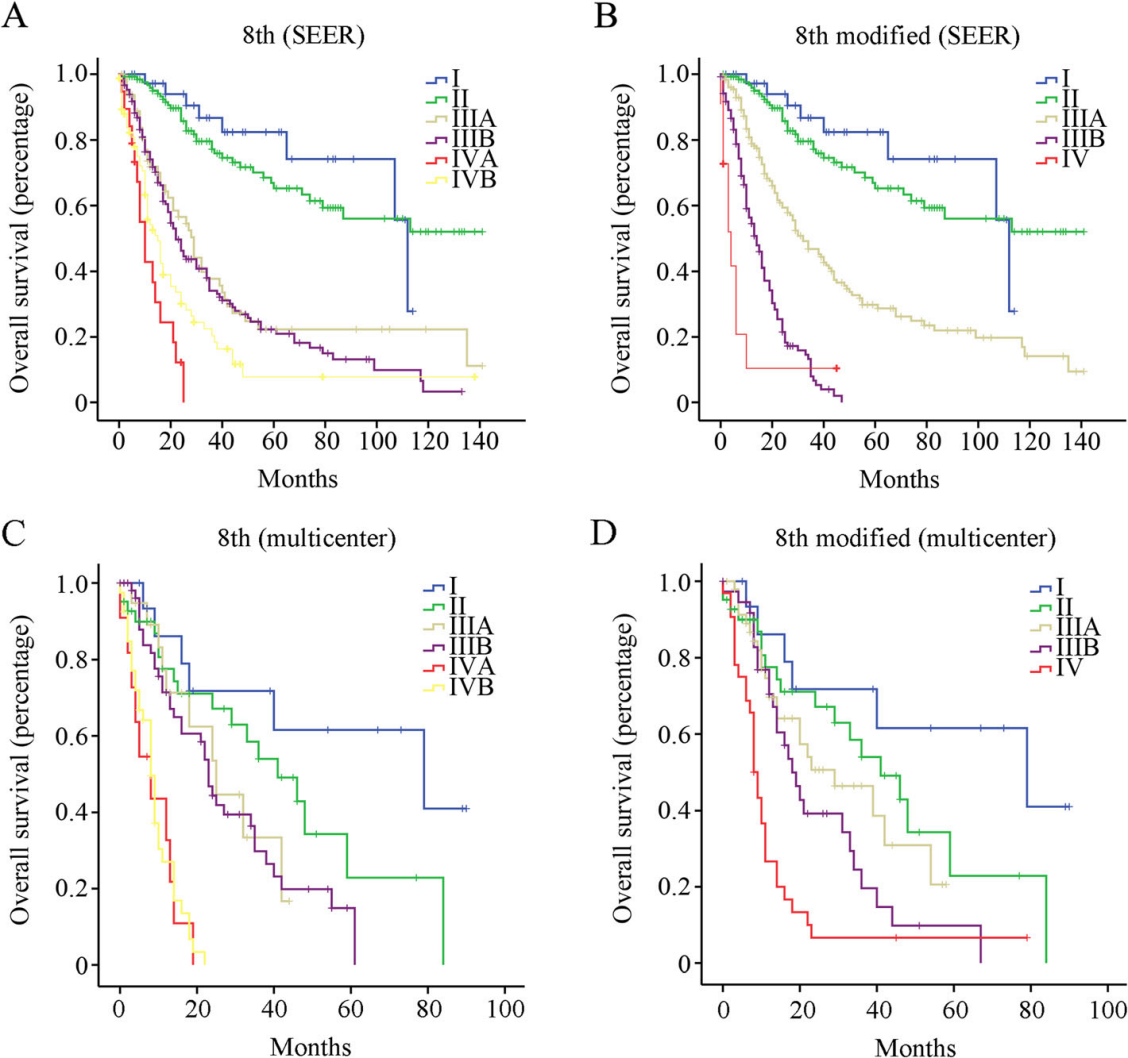
Overall survival (OS) analysis of GBC patients with at least 6 resected LNs. **a** OS of GBC patients from the SEER database using the 8th edition of AJCC staging system. **b** OS of GBC patients from the SEER database using the modified 8th edition of AJCC staging system. **c** OS of GBC patients from the multicenter database using the 8th edition of AJCC staging system. **d** OS of GBC patients from the multicenter database using the modified 8th edition of AJCC staging system

The National Comprehensive Cancer Network (NCCN) guidelines recommend that at least 6 LNs should be examined to ensure the accuracy of staging system. Interestingly, similar results were observed (Fig. 2) when M0 patients with 6 or more examined LNs were selected for further analysis. Indeed, no significant survival difference was observed between stage IIIA and IIIB using the 8th AJCC edition on this subset of patients (*p* > 0.05). In addition, patients with stage IVA showed a worse outcome compared to stage IVB patients, although the difference was not significant. On the other hand, a significant OS difference was observed among stages IIIA, IIIB and IV (*p* < 0.001) using our modified 8th edition on M0 patients with 6 or more examined LNs. For the SEER database, the C-index of the 8th AJCC staging system and our modified staging system were 0.709 (95% CI, 0.622–0.796) and 0.742 (95% CI, 0.655–0.829), respectively. For the multicenter database, the C-index of 8th AJCC staging system was 0.635 (95% CI, 0.591–0.683), while that of our modified staging system was 0.679 (95% CI, 0.604–0.711).

## Discussion

GBC is a highly malignant cancer that is associated with poor prognosis, which underscores the development of a highly sensitive and accurate classification system that can improve disease management and prognosis. AJCC staging system, with its reliable background of evidence-based medicine, has been providing a powerful resource for physicians in their fight against cancer. Compared to the previous edition, the 8th edition of AJCC staging system for GBC included several notable modifications that highlight the critical role of the tumor’s biological characteristics, such as tumor location and LN metastasis, as prognostic indicators of poor outcomes in GBC patients. However, we found that some stages were heterogeneous groups with different long-term outcomes. In this study, we propose modifications to the 8th edition of AJCC staging system for GBC that can improve its discriminatory power and may be considered for inclusion in the next edition of the AJCC staging system.

The shortcomings of the 8th AJCC staging system for GBC are mainly associated with advanced stage cancers, mainly T1–3N1M0 GBC that was regarded as a single prognostic group (stage IIIB). However, our results showed that stage T1–3N1M0 GBC was a heterogeneous group that had better prognostic outcomes than T3N0M0 (stage IIIA). On the other hand, while T1-2N1M0 GBC was prognostically similar to T3N0M0 (*p* = 0.203), it was associated with much better outcomes than T3N1M0 GBC (*p* < 0.001). Consequently, T3N0M0 and T1-2N1M0 were both classified as stage IIIA in the modified staging system. Similar issues were observed in stage IVA and stage IVB.

Similarly, T1-4N2M0 GBC was regarded as a single prognostic group (stage IVB) in the 8th AJCC staging system. However, we observed that T1-4N2M0 GBC was a heterogeneous group that had similar prognosis to stage IVA. Indeed, the median OS of T1-2N2M0 tumors (21 months) was similar to that of T3N0M0 tumors (stage IIIA, 20 months, *p* = 0.667). Similarly, the median OS for T3N2M0 tumors was similar to that of T3N1M0 tumors (11 months vs. 13 months, *p* = 0.186). Furthermore, the median OS of T4N0-1 M0 tumors was similar to that of T3N1-2 M0 tumors (11 months vs. 13 months, *p* = 0.051). Therefore, T3N0M0 and T1-2N1-2 M0 were classified as stage IIIA in the modified version, while T3N1-2 M0 and T4N0-1 M0 were classified as stage IIIB.

The most common route of dissemination of GBC is lymphatic diffusion [8]. While cystic, pericholedochal and hilar LNs are considered the primary route of GBC metastasis, GBC can spread directly to a second (peripancreatic, periduodenal, periportal and perihepatic LNs) or third level (celiac, superior mesenteric artery and the para-aortic LNs) LNs. GBC spreads along the perivascular soft tissue according to the three lymphatic drainage pathways proposed by Ito et al. [9]: cholecysto-retropancreatic pathway (main pathway), cholecysto-celiac and cholecysto-mesenteric pathways (accessory pathways).

The N category was divided into regional LN metastasis (N1) and extra-regional LN metastasis (N2) in the 7th edition of AJCC. The 8th edition adopted a numeric-based system of the N category instead of using the topographic distribution of the positive LNs. This change was supported by evidence showing that the topographic distribution does not represent a correct evaluation of LN status. Indeed, it was demonstrated that GBC with extra-regional LN involvement does not adversely influence the disease specific survival (DSS) like regional LN involvement [10–12]. Besides, Amini et al. [13] showed that the number, but not the location, of positive LNs independently determined the prognosis of GBC after resection. Although using the number of positive LNs might be appropriate for the N staging of GBC, patients with N2 LN metastasis, as defined by the 8th edition of the AJCC staging system, were associated with heterogeneous outcomes. Indeed, not all patients with N2 LN metastasis had a uniformly dismal prognosis. Some patients with N2 LN metastasis were able to achieve a satisfactory survival after radical lymphadenectomy [14, 15]. On the other hand, despite that T4 GBC patients are less likely to receive curative resection than T1–3 GBC patients, it would not be appropriate to categorize all T4 GBC into stage IV [16]. Indeed, patients with T4 GBC with low number of positive LNs (less than 4) can be associated with a relatively good prognosis. Altogether, these findings suggest that modification of the 8th staging system can offer more treatment options and benefits for patients with advanced stage GBC.

LN evaluation could be the most critical step in prognostic assessment. Previous studies have showed that the number of evaluated LNs was closely related to OS. Fan et al. [17] showed that different optimal LN numbers should be assessed for different stages of GBC, with 4, 4 or 5, and 6 LNs being optimal for stages I, II and III A, respectively. They emphasized that 4–6 LNs were related to longer OS in the entire cohort. In addition, Tsilimigras DI et al. [18] reported similar results, where he showed that 4–7 LNs could be the optimal number to evaluate. The 8th edition of AJCC staging system recommended that at least six LNs be assessed. In our study, we observed similar survival plots for the entire cohort after selecting M0 patients with at least 6 examined LNs.

According to Fig. 1, there could be a huge survival gap between early stage (stages I-II) and advanced stages (stages III-IV). Indeed, the median OS of stages I-II was 87 months, which was much longer than that of stages III-IV with a median OS of 14 months (*p* < 0.001). Nevertheless, the modified staging system couldn’t improve the “uneven distribution”, which could be related to the fundamentally different biological behavior between early stage and advanced stage cancers. Interestingly, Yang et al. [19] reported that the low expression of microRNA-125b was lower in advanced stage GBC patients compared to early stage patients, and was significantly associated with patient outcomes.

There are some limitations to this study which should be noted. First, this was a retrospective analysis with low granularity. Second, SEER is a registry that misses a lot of variables such as detailed surgical approaches, residual tumor status (R0, R1 or R2) and postoperative therapy. Third, the new recommendation by AJCC that at least 6 LNs should be assessed has resulted in a lower proportion of patients with six or more LNs resected. Fourth, our conclusions need to be validated by external data. Nevertheless, our novel findings provide insights that can improve the current AJCC staging system for GBC.

## Conclusion

The modified 8th staging system proposed in this study can improve the prognostic precision of the 8th AJCC staging system for GBC. We therefore suggest including these modifications in the next update of AJCC staging system for GBC.

## Supplementary Information

**Additional file 1: Supplement 1.**

## Data Availability

The datasets generated and analyzed during the current study are available from the corresponding author on reasonable request.

## Abbreviations

AJCC: American Joint Committee on Cancer
GBC: Gallbladder carcinoma
SEER: Surveillance, Epidemiology and End Results
C-index: Concordance index
LNs: Lymph nodes
PDAC: Pancreatic ductal adenocarcinoma
OS: Overall survival
DSS: Disease specific survival
