# Supplementary figures and images for: Modification of the 8th American Joint Committee on Cancer staging system for gallbladder carcinoma to improve prognostic precision
Source: BMC Cancer. 2020 Nov 23;20:1129. doi: 10.1186/s12885-020-07578-7 (PMC7682115; doi:10.1186/s12885-020-07578-7)

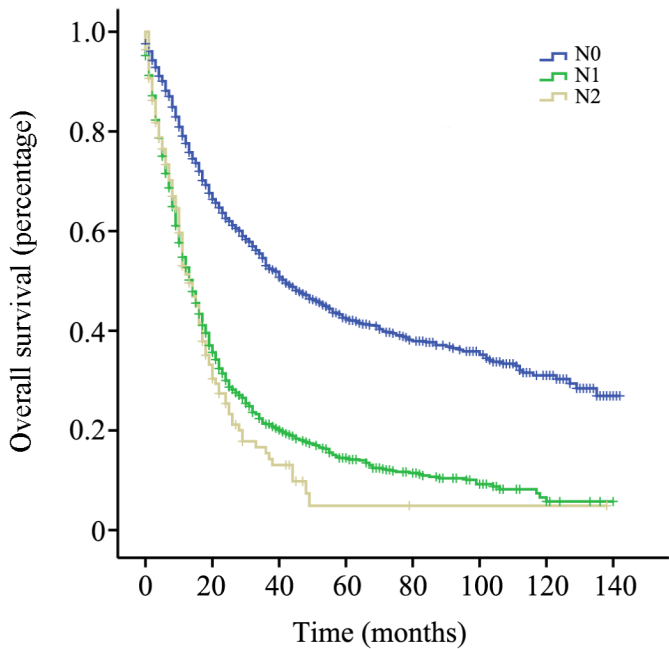

Supplement: Supplementary file 1 — Additional file 1: Supplement 1. [file 12885_2020_7578_MOESM1_ESM.pdf]
